# Does Agricultural Credit Input Promote Agricultural Green Total Factor Productivity? Evidence from Spatial Panel Data of 30 Provinces in China

**DOI:** 10.3390/ijerph20010529

**Published:** 2022-12-28

**Authors:** Fuwei Wang, Lei Du, Minghua Tian

**Affiliations:** School of Economics and Management, Beijing Forestry University, Beijing 100083, China

**Keywords:** agricultural credit input, agricultural green total factor productivity (AGTFP), spatial econometric model, spillover effect, heterogeneity, spatial Durbin model

## Abstract

Improving agricultural green total factor productivity is crucial to promoting high-quality agricultural development. This paper selects the panel data of 30 provinces in China from 2009 to 2020 and uses the super-efficiency SBM model with undesirable outputs to measure the agricultural green total factor productivity of all regions in China. On this basis, this paper uses the panel data fixed-effect model and spatial Durbin model to empirically discuss the impact of agricultural credit input on agricultural green total factor productivity and its spatial spillover effect. The main conclusions are as follows: First, from 2009 to 2020, the average values of agricultural green total factor productivity in national, eastern, central, and western regions are 0.8909, 0.9977, 0.9231, and 0.8068, respectively, and the agricultural green total factor productivity needs to be further improved. Second, the agricultural green total factor productivity presents a significant and positive spatial correlation, and the spatial distribution of agricultural green total factor productivity is not random and irregular. Third, agricultural credit input can significantly promote agricultural green total factor productivity in the local region, but it hinders the improvement of agricultural green total factor productivity in the adjacent regions. Fourth, the impact of agricultural credit input on the agricultural green total factor productivity and its spillover effect has a significant regional heterogeneity. This paper believes that paying attention to the spatial spillover effect of agricultural total factor productivity, optimizing the structure and scale of agricultural credit input, and formulating reasonable agricultural credit policies can improve agricultural green total factor productivity.

## 1. Introduction

Since the reform and opening up, China’s total agricultural and grain output has grown rapidly, and agricultural total factor productivity (TFP) has been improved steadily, which plays an essential role in agricultural development around the world [1]. However, for a long time, the extensive agricultural development model of “high input, high consumption, and high emissions” has resulted in serious environmental pollution problems to some degree with the rapid growth of agricultural TFP [2]. For example, agricultural machinery, fertilizers, pesticides, and agricultural film are essential production inputs in the agricultural production process. On the one hand, increasing these inputs can promote the agricultural TFP. On the other hand, it also produces a lot of carbon emissions and non-point source pollution [2,3]. To solve negative issues on agricultural development and environmental pollution, the Chinese government has advocated a high-quality and green sustainable development mode of agriculture in recent years [4,5]. In 2022, the No. 1 central document of the central government also again emphasized the importance of “promoting green agricultural development, strengthening the comprehensive treatment of agricultural pollution, and improving the efficiency of agricultural resource utilization”. Therefore, when studying the problem of agricultural TFP, it is necessary to focus on agricultural green total factor productivity (AGTFP) that covers environmental constraints [6]. Undoubtedly, improving agricultural green total factor productivity can effectively reduce the environmental pollution in agricultural production [7]. It could be a crucial way to achieve China’s high-quality agricultural and sustainable green development.

How to improve China’s agricultural green total factor productivity? Generally, agricultural credit is likely to be a strong support to agricultural green production activities [8]. Agricultural credit is the general term for credit activities of financial organizations in rural areas including savings and loan businesses. It is a form of mobilizing and distributing temporarily idle monetary funds in rural areas to supply the capital turnover needed by agricultural reproduction [9]. In recent years, the Chinese government has increased its investment in agricultural credit. According to the official data released by the People’s Bank of China, the balance of China’s agricultural loans in 2010 was CNY 11,767.910 billion, which increased to CNY 38,949.3 billion in 2020, with a growth rate of 230.98%. The purpose of increasing the agricultural credit input is to provide a financial guarantee for agricultural development, which can promote the high-quality and green sustainable development of agriculture. Hence, does agricultural credit input effectively promote agricultural green total factor productivity? This is the first question to be discussed in this paper. In addition, China has a vast territory. The trans-regional flow of agricultural production factors has probably caused the phenomenon of spatial spillover in the process of agricultural development in various regions. Therefore, it could be worth investigating, as the second question in this paper, the following: does the impact of agricultural credit input on agricultural green total factor productivity have a spatial spillover effect? Under the background of promoting the high-quality development of China’s agriculture, in-depth research on the impact of agricultural credit input on agricultural green total factor productivity and its spatial spillover effect will have essential theoretical and practical significance for formulating agricultural policies, improving agricultural green total factor productivity, and promoting the green sustainable development of agriculture.

The rest of the paper is structured as follows: Section 2 is the literature review; Section 3 introduces the method and data of this paper; Section 4 presents the results of the empirical research; Section 5 discusses the empirical results; Section 6 introduces the main conclusions and policy implications of this paper.

## 2. Literature Review

Based on the existing research, scholars mainly use the stochastic frontier analysis (SFA) and data envelopment analysis (DEA) to measure the AGTFP. The SFA model is a parameter analysis method that needs to set up a specific form of production function and make strict assumptions [10]. If there are errors in setting production functions and assumptions, the estimation results could be inconsistent with the actual situation [11,12]. Therefore, scholars rarely use the SFA model to measure the AGTFP [1]. Different from the SFA model, the DEA model is a non-parametric analysis method, which does not need to assume the production function’s specific form strictly, and can simulate the productivity of multiple decision-making units using the method of linear programming [13]. Therefore, the DEA model is widely used by scholars to measure the AGTFP [14,15,16].

Among many DEA models, the SBM model and the Malmquist index are common methods to measure the AGTFP. The Malmquist index is a dynamic productivity index, which reflects the moving ratio of the production frontier from the t period to t + 1 period [17,18]. Therefore, many scholars use the Malmquist index to implore the dynamic changes of the AGTFP [6,13,14,19,20]. Although the Malmquist index is widely used to measure the AGTFP, the calculation value is only the moving rate (or change rate) of the production frontier from the t to t + 1 period, not the specific efficiency value. If we want to calculate the DMU-specific efficiency of a year, the Malmquist index is not an ideal method [21]. Different from the Malmquist index, the super-efficiency SBM model-proposed tone [22] can calculate the specific efficiency value of the AGTFP every year. The efficiency value measured by the super-efficiency SBM model does not have the problem of censored data, and therefore has incomparable advantages compared with other models [1]. Therefore, the super-efficiency SBM model has been widely used to study the AGTFP [1,14,15,23,24].

In recent years, scholars have gradually paid attention to the factors affecting the AGTFP. Song et al. [20] proposed that climate change can impact the AGTFP significantly. Yu et al. [24] believed that the implementation of a carbon trading pilot policy has a significant promotion effect on the AGTFP. Sun [25] believed that environmental regulation had limited the development of the AGTFP, but agricultural technology innovation can improve the AGTFP. However, few studies have focused on the impact of finance on the AGTFP, especially agricultural credit. From the existing literature on finance, scholars mainly focused on the impact of agricultural insurance, digital inclusive finance, and financial inclusion on the AGTFP. For example, Fang et al. [26] and Li et al. [27] believed that agricultural insurance positively impacts the AGTFP. Gao et al. [28] indicated that digital inclusive finance mainly promotes the AGTFP by improving green technology. Hu et al. [4] proposed that financial inclusion is a significant driver of the AGTFP.

The existing literature has laid an excellent theoretical foundation for this paper. However, there are still some limitations: Firstly, from the research perspective, the existing literature rarely involves the impact of agricultural credit on the AGTFP and its spatial spillover effect. Secondly, from the perspective of research content, few scholars discuss the influencing factors of the AGTFP and its spatial spillover effect. Thirdly, in terms of research methods, most studies only use the Malmquist index to calculate the dynamic change of the AGTFP and do not consider its influencing factors in depth. In addition, the existing research seldom uses spatial econometric techniques to study the AGTFP and its spatial spillover effects.

To sum up, the marginal contribution of this paper can be summarized in the following four points. Firstly, this paper brings the agricultural credit input and AGTFP into the same analysis framework for discussion. Secondly, based on the panel data of 30 provinces in China, the super-efficiency SBM model with undesirable output is used to measure the AGTFP, which can calculate the specific efficiency value of each DMU. Thirdly, the spatial econometric techniques are used to discuss the impact of agricultural credit input on the AGTFP and its spillover effect, which can solve the problem that the OLS model ignores the spatial spillover effect, thus improving the robustness of the results. Fourthly, according to the regional differences in China, this paper discusses the regional heterogeneity of the impact of agricultural credit input on the AGTFP, making the results closer to the actual situation in China.

## 3. Methods and Data

### 3.1. Spatial Econometric Model

#### 3.1.1. Setting of Spatial Weight Matrix (W)

The spatial weight matrix includes the following three types: the 0–1 weight matrix, the geographic distance weight matrix, and the economic weight matrix. Different weight matrices represent different meanings.

Firstly, the 0–1 weight matrix (*W*_1_) represents the adjacency relationship between regions. If the administrative boundaries of two regions are adjacent, the value is assigned as 1; otherwise, the value is assigned as 0. The mathematical expression of the 0–1 weight matrix is shown as follows:

(1) $$W_{1}=\left\{\begin{array}{l} 1\;\mathrm{if}\;i\;\mathrm{and}\;j\;\mathrm{are}\;\mathrm{adjacent}\\ 0\;\mathrm{otherwise} \end{array}\right.$$

Secondly, the geographical distance weight matrix (*W*_2_) represents the geographical distance between two regions. It is usually calculated by the reciprocal of the square of the actual geographical distance between the two regions. The specific expression is shown in Formula (2) below.

(2) $$W_{2}=\left\{\begin{array}{l} \frac{1}{d^{2}}\;i\neq j\\ 0\;i=j \end{array}\right.\;\mathrm{where},\;i=1\cdots n;j=1\cdots n;n=30$$

Finally, the economic weight matrix (*W*_3_) is calculated based on the level of economic development difference between the two regions. Although some regions do not have adjacency in the geographical location due to the similar level of economic development, the ‘learning and imitation effect’ is likely to make the economic variables in different regions interact [29]. Therefore, the economic weight matrix is often used in spatial econometric analysis. The mathematical expression of the economic weight matrix is shown as follows:

(3) $$W_{3}=\left\{\begin{array}{l} \frac{1}{\left|\bar{GDP_{i}}-\bar{GDP_{j}}\right|}\;i\neq j\\ 0\;i=j \end{array}\right.\;\mathrm{where},i=1\cdots n;j=1\cdots n;n=30$$

Since the spatial spillover effect of agricultural production is more manifested between adjacent provinces, the 0–1 weight matrix (*W*_1_) is selected as the spatial weight matrix of the benchmark regression model. The geographic distance matrix (*W*_2_) and economic weight matrix (*W*_3_) are used for the robustness test.

#### 3.1.2. Spatial Correlation Test

Using the spatial econometric model to analyze problems usually requires a spatial correlation test. The spatial econometric model can be used for further analysis if the test is passed. If the test is not passed, the traditional panel data model can be used for analysis. The Moran index is a common spatial correlation test method, including the Global Moran index and Local Moran index.

It is reasonable to use the Global Moran index to identify the spatial correlation of the AGTFP in different regions. The calculation method is as follows:

(4) $$Global\;Moran’s\;I_{it}=\frac{\sum_{i=1}^{n}\sum_{j=1}^{n}W_{ij}(AGTFP_{i}-\bar{AGTFP})(AGTFP_{j}-\bar{AGTFP})}{S^{2}\sum_{i=1}^{n}\sum_{j=1}^{n}W_{ij}}$$

In Formula (4), *S*^2^ means the sample variance of the AGTFP, and *W_ij_* is the 0–1 weight matrix. The value range of the Global Moran index is between −1 and 1. When the Global Moran’s *I* > 0, it indicates that the AGTFP shows a positive spatial correlation. On the contrary, the Global Moran’s *I* < 0 means that the AGTFP shows a negative spatial correlation. If the Global Moran’s *I* = 0, it indicates no spatial correlation in the AGTFP.

The Local Moran index usually examines whether observations of the AGTFP in different regions have the characteristics of spatial agglomeration. The calculation method is as follows:

(5) $$Local\;Moran’s\;I_{it}=\frac{\left(AGTFP_{i}-\bar{AGTFP})\sum_{j=1}^{n}W_{ij}(AGTFP_{j}-\bar{AGTFP}\right)}{\sum_{i=1}^{n}{\left(AGTFP_{i}-\bar{AGTFP}\right)}^{2}/n}$$

The meanings of all symbols in Formula (5) are the same as above. We usually use the Moran scatter diagram to represent the calculation results of Local Moran’s *I*. The horizontal axis represents the current value of the sample variable, and the vertical axis represents the spatial lag term. The four quadrants of the graph divide the spatial correlation between the sample region and its neighboring regions into four relationships: “high-high” (HH), “high-low” (HL), “low-low” (LL), and “low-high” (LH). Specifically, “high-high” (HH) and “low-low” (LL) indicate that there is a significant spatial positive correlation between the research samples, which means the sample region is a high value (low value), and the surrounding region is also a high value (low value); The “high-low” (HL) and “low-high” (LH) indicate that there is a significant negative spatial correlation between the samples, which means the sample region is a high value (low value), but the surrounding region is a low value (high value).

#### 3.1.3. Model Established

At present, China is at an important stage of comprehensively promoting rural revitalization. Therefore, discussing the impact of the agricultural credit input on the AGTFP can provide a potential academic support for related government decision making. According to the research of Liu et al. [1], the first step is to build the following panel data model to test the impact of agricultural credit input on the AGTFP.

(6) $$AGTFP_{it}=\alpha +\beta \ln Fin_{it}+\delta X_{it}+\mu_{i}+\lambda_{t}+\varepsilon_{it}$$

In Formula (6), AGTFP_it_ represents the agricultural green total factor productivity of province *i* in year *t*, which is the explained variable in this paper. ln*Fin_it_* refers to the agricultural credit input of province *i* in year *t*, which is the core explanatory variable of this paper. *X_it_* is a set of control variables. $\mu_{i}$ is the fixed effect, $\lambda_{t}$ is the time effect, and $\varepsilon_{it}$ is the random disturbance term. *α*, *β*, and *δ* are the regression coefficients of the intercept term, core explanatory variable, and control variable, respectively.

The traditional panel data model usually ignores the possible spatial correlation between variables. However, the spatial econometric model assumes that the variables are interrelated between regions [30]. Therefore, the spatial weight matrix and the spatial lag term are added when setting the spatial econometric model [31]. Spatial econometric models include the spatial autocorrelation model (SAR), spatial error model (SEM), and spatial Durbin model (SDM) [32,33]. In order to discuss the impact of agricultural credit input on the AGTFP and its possible spatial spillover effect, the following spatial econometric model can be proposed.

(7) $$AGTFP_{it}=\rho \sum W_{ij}\times AGTFP_{it}+\alpha \ln Fin_{it}+\delta X_{it}+\varepsilon_{it}$$

(8) $$AGTFP_{it}=\alpha \ln Fin_{it}+\delta X_{it}+\varepsilon_{it}\;,\mathrm{where}\;\varepsilon_{it}=\sigma \sum W_{ij}\times \varepsilon_{it}+\mu_{it}$$

(9) $$AGTFP_{it}=\rho {\sum W_{ij}\times AGTFP_{it}+\alpha \ln Fin_{it}+\delta X_{it}+\tau \sum W_{ij}\times \ln Fin}_{it}+\theta \sum W_{ij}\times X_{it}+\varepsilon_{it}$$

Formulas (7)–(9) are SAR, SEM, and SDM models, respectively. Among them, *ρ* is the spatial lag regression coefficient of the AGTFP, *σ* is the spatial error lag regression coefficient, *τ* is the spatial lag regression coefficient of agricultural credit input, and *θ* is the spatial lag regression coefficient of the control variables. *W* represents the spatial weight matrix. The SAR model represented by Formula (7) only contains the spatial lag term of the explained variable, indicating that the SAR model only considers the spatial spillover effect of the AGTFP. The SEM model represented by Formula (8) only covers the spatial lag term of the random disturbance term. The spatial Durbin model represented by Formula (9) contains both the spatial lag term of the explained variable and the spatial lag term of the core explanatory variable. Therefore, the spatial Durbin model can be used to discuss the spatial spillover effects of the AGTFP and agricultural credit input. However, whether SAR, SEM, or SDM models are selected for analysis needs to be judged through the likelihood-ratio test (LR) [33].

Although the spatial Durbin model covers the spatial and non-spatial correlation terms of sample variables, it does not fully reflect the spatial effects [34]. Therefore, in order to analyze the entire impact paths of agricultural credit input on the AGTFP, this paper adopts the method of a partial differential equation to divide the impact of agricultural credit input on the AGTFP into direct effect, indirect effect, and total effect. The direct effect refers to the influence of explanatory variables on the explained variables in the local province. The indirect effect, also called the spillover effect, measures the degree of the explanatory variable of the local province that affects the explained variable of the adjacent provinces. The total effect is the sum of the direct effect and indirect effect. The general form of the SDM model is shown in Formula (10):

(10) $$Y={\left(I-\rho W\right)}^{-1}nl_{n}+{\left(I-\rho W\right)}^{-1}(X\beta +WX\gamma )+AZ+{\left(I-\rho W\right)}^{-1}\varepsilon$$

For the partial differential equation of the *k*-th explanatory variable of the explanatory variable vector *Y* in Formula (10), we can obtain Formula (11).

(11) $$(\frac{\partial Y}{\partial X_{1k}}\;\frac{\partial Y}{\partial X_{2k}}\;\cdots \;\frac{\partial Y}{\partial X_{nk}})=\left[\begin{array}{ccc} \frac{\partial Y_{1}}{\partial X_{1k}} & \cdots & \frac{\partial Y_{1}}{\partial X_{nk}}\\ \vdots & \ddots & \vdots \\ \frac{\partial Y_{n}}{\partial X_{1k}} & \cdots & \frac{\partial Y_{n}}{\partial X_{nk}} \end{array}\right]={\left(I-\rho W\right)}^{-1}\left[\begin{array}{cccc} \beta_{k} & \omega_{12}\gamma_{k} & \cdots & \omega_{1n}\gamma_{k}\\ \omega_{21}\gamma_{k} & \beta_{k} & \cdots & \omega_{2n}\gamma_{k}\\ \vdots & \vdots & \ddots & \vdots \\ \omega_{n1}\gamma_{k} & \omega_{n2}\gamma_{k} & \cdots & \beta_{k} \end{array}\right]$$

In Formula (11), the direct effect of the *k*-th explanatory variable is the average value of each element of the main diagonal in the matrix. The *k*-th explanation changes the indirect effect into the average value $\frac{1}{n^{2}}\sum_{i=1}^{n}\sum_{j=1}^{n}\omega_{ij}\gamma_{k}$ of all elements in the matrix except the main diagonal element.

### 3.2. Variables

#### 3.2.1. Explained Variable: AGTFP

The agricultural green total factor productivity (AGTFP) is the explained variable of this paper. According to Tone [22,35] and Liu et al. [1], the super-efficiency SBM model with undesirable outputs will be used to calculate the AGTFP. 

Suppose there are *n* decision-making units (DMU), and each DMU includes input vector $x\in {\mathbb{R}}_{+}^{m}$, desirable output vector $y^{g}\in {\mathbb{R}}_{+}^{s_{1}}$, and undesirable output vector $y^{b}\in {\mathbb{R}}_{+}^{s_{2}}$, respectively. Then, we can define the matrices of input vector, desirable output vector, and undesirable output vector as $X=\left[x_{1},x_{2}\cdots ,x_{n}\right]\in {\mathbb{R}}_{+}^{m\times n}$, $Y^{g}=\left[y_{1}^{g},y_{2}^{g},\cdots ,y_{n}^{g}\right]\in {\mathbb{R}}_{+}^{s_{1}\times n}$, and $Y^{b}=\left[y_{1}^{b},y_{2}^{b},\cdots ,y_{n}^{b}\right]\in {\mathbb{R}}_{+}^{s_{2}\times n}$, respectively. On this basis, the production possibility set (PPS) can be defined as: $PPS=\left\{(x,y^{g},y^{b})\left|x\geq X\lambda ,y^{g}\leq Y^{g},y^{b}\geq Y^{b},\lambda \geq 0\right.\right\}$ where *λ* is the non-negative intensity vector. In summary, the algorithm of the super-efficiency SBM model with undesirable outputs is shown in Formula (12).


(12) $$\begin{array}{ll} & \min\rho =\frac{1+\frac{1}{m}\sum_{i=1}^{m}\frac{s_{i}^{-}}{x_{ik}}}{1-\frac{1}{s_{1}+s_{2}}\left(\sum_{r=1}^{s_{1}}\frac{s_{r}^{g}}{y_{rk}^{g}}+\sum_{t=1}^{s_{2}}\frac{s_{t}^{b}}{y_{tk}^{b}}\right)}\\ s.t. & \sum_{j=1,j\neq k}^{n}x_{ij}\lambda_{j}-s_{i}^{-}\leq x_{ik}\\ & \sum_{j=1,j\neq k}^{n}y_{rk}\lambda_{j}+s_{r}^{g}\geq y_{rk}^{g}\\ & \sum_{j=1,j\neq k}^{n}y_{tj}\lambda_{j}-s_{t}^{b}\leq y_{tk}^{b}\\ & \sum_{j=1,j\neq k}^{n}\lambda_{j}=1\\ & \lambda \geq 0,s_{i}^{g}\geq 0,s_{r}^{b}\geq 0,s^{-}\geq 0\\ & i=1,2,\cdots m;r=1,2,\cdots s_{1};t=1,2,\cdots s_{2};j=1,2,\cdots n(j\neq k) \end{array}$$

In Formula (12), the slack variable of inputs, desirable outputs, and undesirable outputs are $s_{i}^{-}$, $s_{r}^{g}$, and $s_{r}^{b}$, respectively; $\lambda_{j}$ is the weight vector; *ρ* is the efficiency value of DMU, and when *ρ* > 1, it signifies the DMU is effective.

According to Du et al. [12] and Yu et al. [24], we have built an input–output indicators system and use the super-efficiency SBM model with undesirable output to measure the AGTFP. The specific contents of the indicators are shown in Table 1.

The agricultural input contains labor, mechanics, land, water, power, fertilizer, pesticide, and plastic sheeting. The agricultural output contains the desirable output and undesirable output. Among them, the desirable output is represented by the gross value of agriculture (units: CNY 10 thousand), and the undesirable output is represented by the agricultural carbon emissions (units: 10,000 tons). Carbon emission is the main factor affecting the climate and environment and can be used to characterize pollutants in agricultural production [2,24]. Therefore, it is reasonable to use agricultural carbon emissions to represent the undesirable output. Agricultural carbon emissions mainly come from six parts: fertilizer, pesticides, agricultural plastic sheeting, agricultural diesel, agricultural ploughing, and agricultural irrigation, respectively [1,2,24]. We can use Formula (13) to calculate the agricultural emissions.

(13) $$C=\sum C_{r}=\sum S_{r}•\delta_{r}$$

where *C* is the agricultural total carbon emissions, *C_r_* refers to the carbon emissions from agricultural carbon emission sources, *S_r_* refers to the number of agricultural carbon emission sources, and *δ_r_* represents the coefficient of agricultural carbon emission sources. The carbon emission coefficient of each agricultural carbon emission source is shown in Table 2.

#### 3.2.2. Core Explanatory Variable

The core explanatory variable of this paper is agricultural credit input. In the existing research, scholars mainly use farmers’ loans to characterize agricultural credit input [8,36,37]. However, this indicator only partially reflects the agricultural credit input. As agricultural credit covers a relatively wide range, it is necessary to describe the data information contained in this concept as much as possible. Since 2009, the China Financial Yearbook and China Rural Financial Development Report have published the balance of agricultural loans of financial institutions, which are mainly divided based on the purpose, urban and rural regions, and the loan subject. Specifically, they cover loans for agriculture, forestry, animal husbandry and fishery, agricultural materials and agricultural by-products circulation, rural infrastructure construction, rural (county and below) loans, rural enterprises, and various organizations. Compared with the indicator of farmers’ loans, the balance of the agricultural loans of financial institutions contains more extensive content. It can better reflect the support of agricultural credit to the agricultural economy and development. Therefore, this paper chooses the balance of agricultural loans of financial institutions as the alternative indicator of agricultural credit.

#### 3.2.3. Control Variables

In addition to agricultural credit input, many factors can affect agricultural green total factor productivity. According to the studies of Song et al. [20] and Fang et al. [26], we have controlled the other variables that affect the AGTFP in the model. The control variables include industrial structure, agricultural financial expenditure, degree of opening up, urbanization level, economic development level, and disaster area.

**Figure 1:**
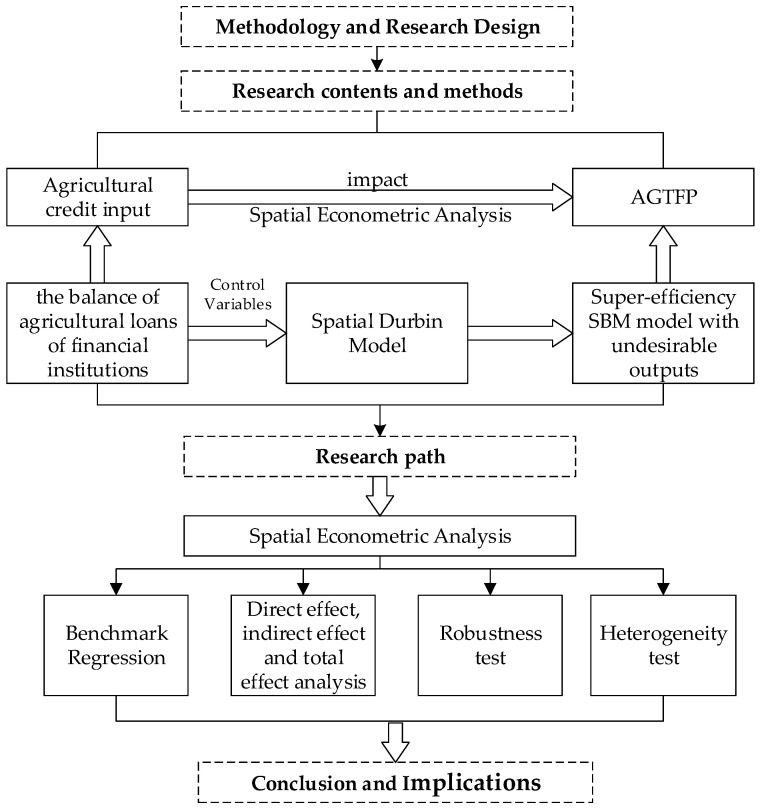
Research roadmap.

To sum up, the research roadmap of this paper is shown in Figure 1. The definitions and symbols of the explained variable, core explanatory variable, and control variables are shown in Table 3.

### 3.3. Study Area, Data Sources, and Statistical Description

**Figure 2:**
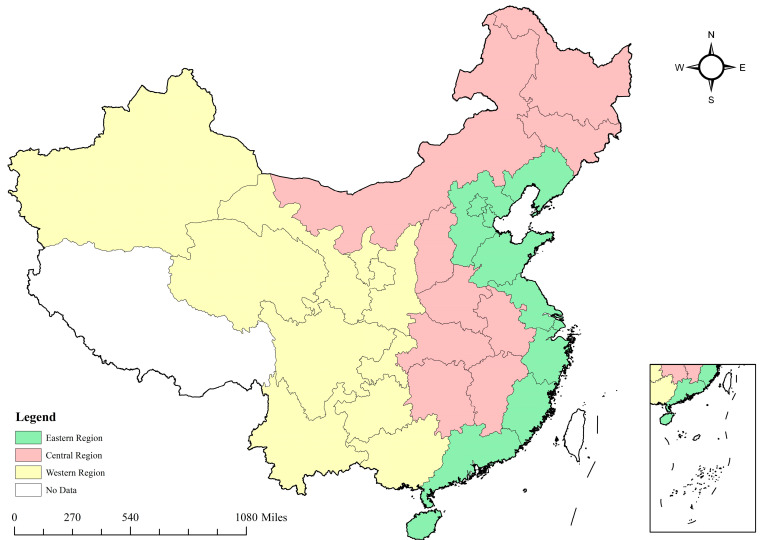
Study area.

The study area of this paper is 30 provinces in mainland China (excluding Hong Kong, Macao, and Taiwan). Due to the severe lack of relevant data, Tibet is excluded from this paper. In addition, China has a large land area, and the drivers behind the AGTFP in different regions are different. Therefore, according to China’s economic development and geographical characteristics, we divide the Chinese mainland into three regions: the eastern region, the central region, and the western region, for further discussion. The study area of this paper is shown in Figure 2.

The original data involved are from the official data published in the China Statistical Yearbook, China Rural Statistical Yearbook, China Financial Yearbook, and Wind database. Since the data related to agricultural credit in various regions before 2009 have not been published, considering the availability of data, we select the panel data of 2009–2020. For a small number of missing values, we use linear interpolation to complete the missing values. In addition, to solve the dimension problem of different indicators and improve the authenticity and robustness of the regression results, we make the logarithmic treatment on indicators such as agricultural credit, economic development level, and disaster area during the regression process. The statistical description and the correlation matrix of the study samples are shown in Table 4 and Table 5 below.

## 4. Results

### 4.1. The Calculation Results of AGTFP

**Figure 3:**
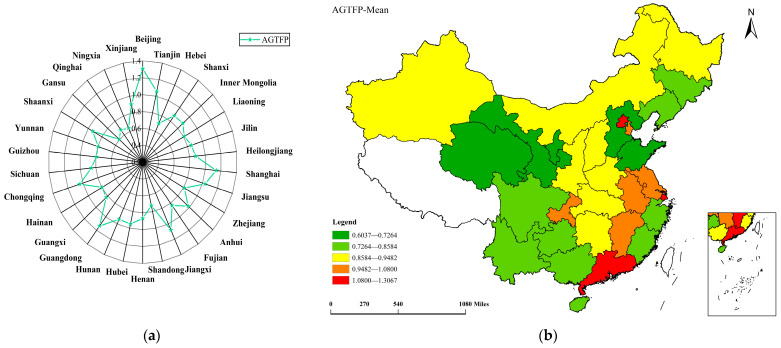
The regional distribution characteristics of AGTFP. (**a**) Radar map of AGTFP mean value of each region; (**b**) spatial map of AGTTP.

**Figure 4:**
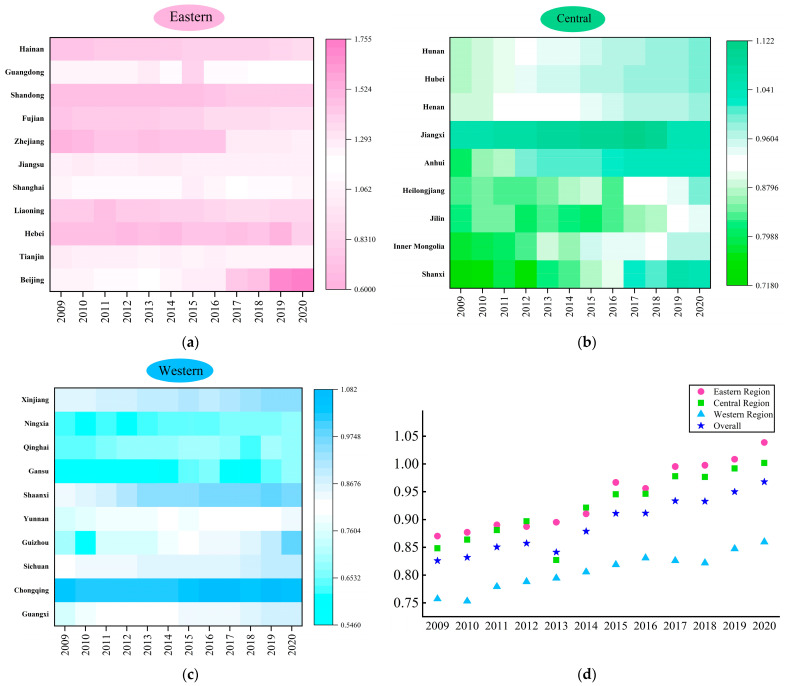
Temporal distribution characteristics of AGTFP. (**a**) Temporal distribution characteristics of AGTFP in eastern region; (**b**) temporal distribution characteristics of AGTFP in central region; (**c**) temporal distribution characteristics of AGTFP in western region; (**d**) temporal distribution characteristics of AGTFP in all regions.

Based on the super-efficiency SBM model with undesirable outputs, we calculated the AGTFP in 2009–2020 using the MaxDEA pro 6.18 software. The calculation results are shown in Figure 3 and Figure 4 below.

Through calculation, we found that the average value of the AGTFP in China from 2009 to 2020 was 0.8909 (AGTFP < 1), which means that the AGTFP in China has yet to reach an effective state, and there is still room for further improvement. Therefore, in future agricultural development, it is necessary to further improve the level of cleaner production and reduce the environmental pollution caused by agricultural production to enhance the AGTFP in China.

Figure 3 describes the regional distribution characteristics of the AGTFP in China. Among the 30 provinces, only 7 regions have AGTFP values greater than 1, reaching the effective frontier, namely, Beijing (1.3067), Shanghai (1.1407), Guangdong (1.1338), Jiangxi (1.0800), Tianjin (1.0551), Chongqing (1.0406), and Jiangsu (1.0331), respectively, and others are less than 1. It is illustrated that most provinces with a high AGTFP are located in the eastern region, while those provinces in the central and western regions have relatively low AGTFP. To some extent, agricultural production in most regions has the problems of high pollution, high emissions, and low efficiency. The level of green agricultural production needs to be further improved.

Figure 4 describes the temporal distribution characteristics of the AGTFP in China. From 2009 to 2020, the AGTFP showed an apparent fluctuating upward trend. The AGTFP value in 2009 was 0.8258, which increased to 0.9678 in 2020, with a growth rate of 17.18%. This shows that the AGTFP has been continuously improved, and more attention has been paid to environmental protection in agricultural production. From the perspective of the regional level, the average values of the AGTFP in the eastern, central, and western regions were 0.9977, 0.9231, and 0.8068, respectively. The AGTFP in the eastern and central regions was higher than the national average, while in the western regions it was lower than the national average. Moreover, the AGTFP in three regions also presented a fluctuating upward trend in temporal characteristics.

### 4.2. Results of Spatial Correlation Test

The calculation results of Global Moran’s I are shown in Table 6. It is illustrated that the Global Moran’s I of the AGTFP in each year are all significant and positive. This indicates that the AGTFP presents a significant and positive spatial correlation.

**Figure 5:**
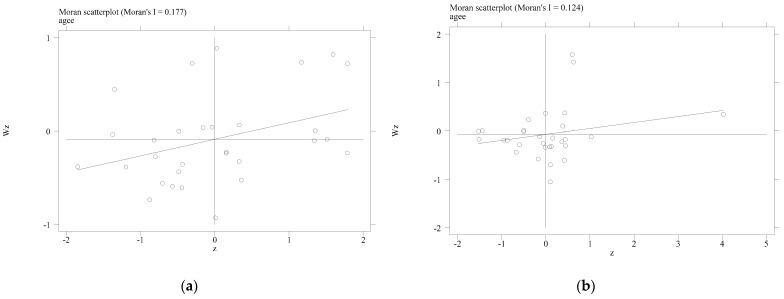
The Moran scatter diagram of AGTFP in 2009 and 2020. (**a**) The Moran scatter diagram of AGTFP in 2009; (**b**) the Moran scatter diagram of AGTFP in 2020.

From the calculation result of the Local Moran’s *I* and the Moran scatter diagram shown in Figure 5, an interesting phenomenon can be proposed that the spatial distribution of the AGTFP is not random and irregular. Most provinces are located in the first and third quadrants, which indicates that the AGTFP shows a prominent spatial agglomeration characteristic. Specifically, the AGTFP shows the spatial distribution characteristics of “high-high” and “low-low”.

To sum up, the AGTFP of each province has a significant spatial correlation, so it is reasonable to use the spatial econometric model for the following analysis.

### 4.3. Results of Benchmark Regression

Before the analysis, we needed to use the LR test and Wald test to verify whether SAR, SEM, or SDM models should be used [34,38]. Table 7 shows the results of the LR and Wald tests under spatial matrices *W*_1_, *W*_2_, and *W*_3_. According to the results, we can find that the *p*-values of the LR and Wald tests were all 0.000, indicating that the SDM model could not degenerate into the SAR and SEM models. Therefore, the SDM model is suitable for further analysis.

To test the impact of agricultural credit input on the AGTFP, we give the benchmark regression results of the panel data model and spatial econometric model in Table 8.

Table 9 shows the results of direct, indirect, and total effects calculated according to Formula (11). It can be found that the results are close to Column (5) in Table 8, which indicates that the regression results are authentic and effective.

In Table 8, Column (1) and Column (2) are the estimated results of the panel data fixed-effect model and random-effect model, respectively. The Hausman test proves that the fixed-effect model is superior to the random-effect model. Therefore, we use the estimation results in Column (1) to explain the impact of agricultural credit input on the AGTFP. In addition, the estimation method of the fixed-effect model is the least square method.

From Column (1), the coefficient of lnFin is 0.0528, which is significant and positive at the level of 5%. It indicates that a 1% increase in agricultural credit input can increase the AGTFP by 0.0528. Namely, the agricultural credit input can significantly promote the AGTFP. For control variables, the coefficients of Open and lnRGDP are 0.0499 and 0.1103, respectively, which are significant and positive at the level of 1%. This means that the degree of opening up and economic development level can improve the AGTFP. The coefficients of Urban and lnDis are −0.5316 and −0.0096, which are significant and negative at the level of 10%. The result indicates that the level of urbanization and natural disasters in a region is not conducive to the improvement of the AGTFP. The regression coefficients of the industry structure and agricultural fiscal expenditure are positive but insignificant.

Furthermore, we use the spatial econometric model to analyze the spatial spillover effect of agricultural credit input on the AGTFP. Columns (3) to (5) in Table 8 are the regression results of SAR, SEM, and SDM, respectively. The spatial econometric model assumes a significant spatial correlation between the variables. Therefore, the spatial econometric model does not conform to the classical assumptions of the OLS model, and the results obtained by using the MLE method may be more accurate.

Based on Columns (3) to (5), it is suggested that the regression coefficients of lnFin on the AGTFP are significant and positive under the spatial econometric model. The results are the same as the result of the fixed-effect model. These results once again prove that agricultural credit input has a significant positive impact on the AGTFP.

In Column (5), the coefficient of the spatial lag term (*ρ*) is 0.4217, which is significant and positive under the significance level of 1%. This result shows that the AGTFP has a significant positive spatial spillover effect. Namely, the improvement of the AGTFP in the local region can drive the development of the AGTFP in the adjacent regions. The regression coefficients of lnFin and W × lnFin are 0.0461 and −0.1688, respectively, which are all significant. This means that the agricultural credit input positively impacts the AGTFP in the local region, but it hinders the improvement of the AGTFP in the surrounding regions.

For control variables in Column (5), the level of urbanization and natural disasters have a negative impact on the AGTFP in the local region. The industry structure in the local region may have a negative impact on the AGTFP in the adjacent regions. The degree of opening up in the local region positively impacts the AGTFP in the adjacent regions.

The direct effect of this paper is to explore the overall impact of agricultural credit input on the AGTFP in the local region. According to Du et al. [29], this overall impact usually includes two aspects. On the one hand, it includes the direct impact on the AGTFP when the agricultural credit input may be changed. On the other hand, the agricultural credit input may affect the AGTFP in the local region through affecting the AGTFP in the surrounding regions, thus generating a “feedback effect”. Therefore, the direct effect is the sum of spatial Durbin model estimation results and the “feedback effect”. Column (1) in Table 9 is the result of the direct effect. The results show that agricultural credit input has a significant and positive impact on the AGTFP in the local region. This is consistent with the conclusion estimated by the spatial Durbin model. The “feedback effect” of agricultural credit input on the AGTFP is 0.0392, which means agricultural credit input can promote the AGTFP in the local region by affecting the AGTFP of other regions.

The indirect effect of this paper indicates the impact of agricultural credit input on the AGTFP in surrounding regions. In other words, the indirect effect represents the spatial spillover effect of agricultural credit input on the AGTFP. Column (2) of Table 9 shows the results of the indirect effect. When the agricultural credit input in the local region increases by 1%, the AGTFP of the surrounding provinces will be decreased by 5.89%. This conclusion verifies again that the agricultural credit input positively impacts the AGTFP in the local region, but it hinders the improvement of the AGTFP in the surrounding regions.

The total effect is equal to the direct effect plus the indirect effect. In Column (3), the total effect is 0.0263. In addition, the estimation results of the direct and indirect effects of each control variable are similar to the benchmark regression results.

### 4.4. Results of Robustness Test

This paper tests the robustness of benchmark regression results by replacing the weight matrix and the core explanatory variables. The results of the robustness test are displayed in Table 10.

Columns (1)–(6) are the estimated results of the replacement weight matrix. This paper uses the geographic distance matrix (*W*_2_) and economic weight matrix (*W*_3_) to replace the 0–1 matrix (*W*_1_) for regression. Column (7) shows the estimated results of replacing the core explanatory variables. This paper uses the balance of regional farmers’ loans to replace the balance of the agricultural loans of financial institutions.

In Table 10, we present the following findings. Firstly, the spatial lag term regression coefficients (*ρ*) are all positive and significant under the different models. This result proves again that the AGTFP in the local region significantly impacts the AGTFP in the adjacent regions. Secondly, the coefficients of lnFin in different models are mostly positive and significant, again proving that the agricultural credit input can improve the AGTFP in the local region. Thirdly, the coefficients of W × lnFin in different models are negative, proving that the agricultural credit input in the local region may have a negative impact on the AGTFP in the surrounding regions. Finally, the direction and significance level of the control variables are roughly the same as the benchmark regression results. Therefore, it is believed that the results of the benchmark regression are robust.

### 4.5. Results of Heterogeneity Test

#### 4.5.1. Results of Regional Heterogeneity Test

This paper divides China into eastern, central, and western regions to further discuss the regional differences in the impact of agricultural credit input on the AGTFP. Table 11 shows the results of the regional heterogeneity test.

According to Table 11, the impact of agricultural credit input on the AGTFP in different regions has an apparent heterogeneity. The coefficients of the spatial lag term in the three regions are all significant and positive. The regression results of lnFin in the eastern, central, and western regions are 0.1847, 0.1311, and 0.0174, respectively. The coefficients show decreased characteristics from eastern to western, that is, eastern region > central region > western region. In addition, the coefficients of lnFin in the eastern and central regions are significant and positive, but the western region is not significant. The regression coefficients of *W* × lnFin in the eastern, central, and western regions are −0.6269, −0.0506, and −0.5576, respectively. The eastern and western regions are significant and negative, and the central region is negative but not significant.

#### 4.5.2. Results of Temporal Heterogeneity Test

Considering that agricultural credit input may have a lagging impact on agricultural green total factor productivity, this paper makes a regression analysis on the lag of agricultural credit input by one period and two periods to further verify the impact of agricultural credit input on the AGTFP. The regression results are shown in Table 12.

In Table 12, Column (1) is the regression result of agricultural credit input lagging behind the one period, and Column (2) is the regression result of agricultural credit input lagging behind the two phases. When the agricultural credit input lags behind for one period, the agricultural credit input significantly promotes the AGTFP in the local region, and significantly inhibits the AGTFP in the surrounding regions. However, when agricultural credit input lags behind two periods, agricultural credit input can only significantly promote the AGTFP in the local region. The impact of the AGTFP in surrounding regions is not significant.

## 5. Discussions

### 5.1. Spatial Distribution and Agglomeration Characteristics of AGTFP

The spatial distribution of the AGTFP in Figure 3 shows the characteristics of “high in the east and low in the west” and gradually decreases from east to west. This result is consistent with the research conclusions of Zhong et al. [13] and Zhu et al. [16]. The temporal distribution characteristics of the AGTFP in Figure 4 show that the AGTFP in China rose annually in fluctuations during 2009–2020. Although the AGTFP grows yearly, it still can be further improved, consistent with the conclusion of Liu et al. [1].

The reasons why the AGTFP shows the characteristics of “high in the east and low in the west” may be as follows. Firstly, the eastern region has high economic development and advanced agricultural green production technology. Moreover, the agricultural development in the eastern region adheres to the strategy of “invigorating agriculture through quality” and “invigorating agriculture through science and technology”. Advanced technology in agricultural production can protect the environment, strengthen resource management, as well as reduce agricultural carbon emissions, which can achieve the unity of agricultural economic growth and ecological environment protection [39]. Secondly, the growth rate in the central and western region is slow, despite showing a fluctuating upward trend. Due to its natural endowment and economic development inertia, the central and western regions rely on using chemicals, fossil energy, and other goods with high carbon emission coefficients in agricultural production. As a result, the carbon sink function of the agricultural ecosystem has been weakened, and is unable to absorb a large amount of agricultural carbon dioxide, which leads to the fact that the overall absorption capacity of agriculture to carbon dioxide has been weakened [23]. Therefore, reducing agricultural carbon emissions could still be an important solution for raising the AGTFP among regions.

From the temporal distribution characteristics of the AGTFP during 2009–2020, we found that the AGTFP grew slowly before 2013 and showed rapid growth after 2013. The main reason for this finding could be the fact that China’s economy entered a new stage of development after 2013. The strategies of reducing poverty, rural revitalization, as well as the progress of green agricultural production technology, etc., have promoted the rapid improvement of the AGTFP.

In addition, the results of spatial correlation tests indicate that the AGTFP presents a significant and positive spatial correlation and shows the characteristics of “high-high” and “low-low” in space. The high-value regions are most distributed in the eastern region, and the low-value regions are most distributed in the central and western regions.

### 5.2. The Impact of Agricultural Credit Input on AGTFP and Its Spatial Spillover Effect

Through the empirical analysis in Section 4, we found that the agricultural credit input can positively impact the AGTFP in the local region. However, it inhibits the improvement of the AGTFP in the adjacent regions. In this section, we will discuss the reasons for this phenomenon.

Firstly, the regression results of the fixed-effect model of panel data and the SDM model indicate that the agricultural credit input can positively impact the AGTFP in the local region. The reasons for the result may be as follows. On the one hand, the increase in agricultural credit input can solve the problem of the capital shortage of agricultural enterprises in technological innovation and promote agricultural green technological innovation. When the latest agricultural technology achievements are applied into agricultural production practice, it can promote the progress of agricultural green production technology, thus promoting the improvement of the AGTFP. On the other hand, agricultural credit input can improve farmers’ scientific and technological literacy and application ability. If it is easier for farmers to obtain credit funds, the agricultural cleaner production capacity will be improved, and so agricultural pollution and carbon emissions will be reduced, the AGTFP can be increasing as well [9]. What is more, agricultural credit input can benefit the allocation efficiency of agricultural input factors. In the traditional mode, farmers often increase outputs via increasing the input of means of production, resulting in environmental pollution. The increase in agricultural credit investment provides a financial guarantee for farmers to introduce cleaner production. Farmers will widely use new equipment and new production technology to improve the agricultural output and reduce the environmental pollution caused by the use of means of production; therefore, the AGTFP improves.

Secondly, the agricultural credit input in the local region can discourage the improvement of the AGTFP in the adjacent regions. Generally, agricultural credit activities only occur within local provinces, and there are relatively few cross-provincial cases. Due to the significant difference in the economic and financial development levels between different regions, the agricultural credit support provided for green agricultural production is also significantly different. However, to obtain a higher capital income, agricultural production factors (i.e., agricultural machinery leasing activities, labor, etc.) usually flow from regions with low agricultural credit to high agricultural credit. This phenomenon is particularly evident in the border region between the two provinces. It will not benefit the AGTFP in regions with low agricultural credit input that some production factors flow into regions with high agricultural credit input.

Thirdly, the impacts of the agricultural credit scale on the AGTFP and its spatial spillover effect are different in the eastern, central, and western regions. Based on the regional heterogeneity test, it is argued that the coefficients of lnFin showed decreased characteristics from the eastern to western regions, namely, eastern region > central region > western region. This indicates that the agricultural credit input has the most significant impact on the AGTFP in the eastern region, followed by the central and western regions. However, the spatial spillover effect of the agricultural credit input in the local region on the AGTFP in the adjacent regions showed a different characteristic. The agricultural credit input in the eastern and western regions significantly inhibited the growth of the AGTFP in the surrounding regions, while that in the central region was not significant. The potential reasons could be as follows. Firstly, the eastern and central regions have a relatively high level of economic development, sufficient agricultural credit funds, and good conditions for green agricultural production. Therefore, agricultural credit input in these two regions has significantly improved the AGTFP in the local region. However, the basic conditions for agricultural production in the western region are underdeveloped in some degree, including insufficient public services and infrastructure that needs to be improved, such as water supply and drainage, roads and scattered plots, and soil conditions. These problems could lead to the fact that the agricultural credit input is mainly used to improve the basic requirements of agricultural production in a short time, so the impact on the AGTFP is insignificant. In addition, most of the central regions are the main grain-producing areas in China. The production conditions and capital situation in the central region are homogeneous. Therefore, the impact of the agricultural credit input on the surrounding areas is not significant in the central region.

## 6. Conclusions and Policy Recommendations

### 6.1. Conclusions

Based on the panel data of 30 provinces in China from 2009 to 2020, this paper uses the super-efficiency SBM model with undesirable outputs to measure China’s agricultural green total factor productivity (AGTFP). On this basis, we use the fixed-effect model of panel data and the spatial econometric model to empirically test the impact of agricultural credit input on the AGTFP and its spatial spillover effect. The main conclusions of this paper are as follows.

Firstly, from 2009 to 2020, the average value of the AGTFP in China was 0.8909, which means that the AGTFP in China has yet to reach an effective state. From the time evolution trend perspective, the AGTFP in China showed an apparent fluctuating upward trend yearly. From the perspective of different regions, the average values of the AGTFP in the eastern, central, and western regions were 0.9977, 0.9231, and 0.8068, respectively (eastern region > central region > western region).

Secondly, the AGTFP presents a significant and positive spatial correlation, and the spatial distribution of the AGTFP is not random and irregular. Regions with similar AGTFP levels have significant spatial agglomeration characteristics.

Thirdly, agricultural credit input can significantly promote the AGTFP in the local region, but it hinders the improvement of the AGTFP in the adjacent regions. The degree of opening up and the economic development level can improve the AGTFP in the local region, and the level of urbanization and natural disasters in a region is not conducive to the AGTFP in the local region.

Fourthly, the impact of agricultural credit input on the AGTFP and its spillover effect has a significant regional heterogeneity. The agricultural credit input has the most significant impact on the AGTFP in the eastern region, followed by the central and western regions. Agricultural credit input in the eastern and western regions significantly inhibits the growth of the AGTFP in the surrounding regions, while that in the central region is not significant.

### 6.2. Policy Recomendations

Based on the above conclusions, this paper proposes the following policy recommendations:

Firstly, there is still a particular gap between the current development level of China’s agricultural green total factor productivity and the goal of high-quality agricultural development. Therefore, to improve agricultural green total factor productivity, we must focus on resource conservation and pollution control while actively developing agricultural production. As the best practitioner of agricultural production, the eastern region should unswervingly strengthen the R&D and promotion of agricultural green science and technology, optimize resource allocation, and achieve the sustainable growth of agricultural green total factor productivity. The agricultural foundation of the central region is between the west and the east, so we should enhance the efficiency of green agricultural technology while improving the green production technology of production agriculture. The western region has apparent advantages of backwardness and has achieved catch-up growth.

Secondly, agricultural green total factor productivity shows an apparent spatial aggregation, and each province can be affected by the neighboring provinces to a certain extent. Therefore, it is necessary to pay special attention to the driving role of provinces with high agricultural green total factor productivity levels in the surrounding regions. We should strengthen the interaction of agricultural production in neighboring areas; promote the rational flow of agricultural technology, talents, capital, and other production factors among regions; and strengthen the information flow and technology sharing of agricultural production among regions.

Thirdly, we should expand the scale of agricultural credit investment. Increasing the agricultural credit input can effectively improve the agricultural green total factor productivity in the local region. Therefore, all provinces should improve the agricultural credit input mechanism and pay attention to the efficiency of agricultural credit while ensuring the stable expansion of the agricultural credit scale. Meanwhile, financial institutions should optimize the structure of agricultural credit input and continue to increase financial support in the agricultural green innovation field to promote the improvement of agricultural green total factor productivity. In addition, it is necessary to improve the corresponding laws and regulations to provide legal protection for agricultural credit investment to promote the AGTFP.

Fourthly, according to local conditions, it is necessary to formulate agricultural credit policies consistent with the improvement of green total factor productivity in the region. The higher level of agricultural credit may inhibit improving agricultural green total factor productivity in surrounding areas. Therefore, when formulating agricultural credit policies, the government and financial institutions need to reasonably formulate relevant policies from the actual situation of the region to promote the coordinated improvement of agricultural green total factor productivity.

Due to the limitations of the research, this paper may also have the following deficiencies, which are expected to be improved in further research in the future. Firstly, the data can be further updated to observe the latest changes in the AGTFP. Secondly, it is also interesting to use city- or county-level data to measure the AGTFP and consider its influencing factors. In a word, we hope this paper can promote more research in the field of agricultural credit input and agricultural green total factor productivity and design some policies to improve agricultural green total factor productivity.

## Figures and Tables

**Table 1:** The input–output indicators system of AGTFP.

Indicator’s Type	Indicator’s Name	Meaning	Units
Input	Labor	The number of agricultural employees	10 thousand people
	Mechanics	The total power of agricultural machinery	10,000 kw
	Land	The total planting area of crops	1000 HA
	Water	Effective irrigation area	1000 HA
	Power	Agricultural diesel consumption	10,000 tons
	Fertilizer	The amount of pure fertilizer application	10,000 tons
	Pesticide	The use amount of pesticide	10,000 tons
	Plastic sheeting	Consumption of agricultural plastic film	10,000 tons
Desirable output	GDP	Gross value of agriculture	10,000 yuan
Undesirable output	Agricultural total carbon emissions	$C=\sum C_{r}=\sum S_{r}•\delta_{r}$	10,000 tons

**Table 2:** Agricultural carbon emission source, coefficient, and reference.

Carbon Source	Carbon Emission Coefficient	Reference
Fertilizer	0.8956 kg·kg^−1^	Oak Ridge National Laboratory, ORNL
Pesticides	4.9341 kg·kg^−1^	Oak Ridge National Laboratory, ORNL
Agricultural plastic sheeting	5.18 kg·kg^−1^	Institute of Resources, Ecosystem and Environment of Agriculture, IREEA, Nanjing Agricultural University
Agricultural diesel	0.5927 kg·kg^−1^	IPCC
Agricultural ploughing	312.6 kg·kg^−2^	Institute of Agriculture and Biotechnology of China Agricultural University, IABCAU
Agricultural irrigation	20.476 kg/hm^2^	Li et al. (2011)

**Table 3:** The definitions and symbols of all variables.

Type	Variables	Symbol	Definitions
Explained variable	Agricultural green total factor productivity	AGTFP	The calculation value of AGTFP based on the super-efficiency SBM model with undesirable outputs
Core explanatory variable	Agricultural credit input	lnFin	The balance of agricultural loans of financial institutions
Control variables	Industrial structure	IS	The proportion of added value of the primary industry in GDP
	Agricultural fiscal expenditure	FE	The proportion of agricultural expenditure in total financial expenditure
	Degree of opening up	Open	The proportion of total foreign direct investment in GDP
	Urbanization level	Urban	The proportion of urban population in total population
	Economic development level	lnRGDP	Per capita GDP
	Disaster area	lnDis	Affected area of crops

**Table 4:** Statistical description of the sample.

Variables	Obs	Mean	Std.Dev	Min	Max
AGTFP	360	0.8932	0.1704	0.5464	1.7504
lnFin	360	8.5793	0.9336	5.8886	10.7557
IS	360	0.9996	0.0558	0.0027	0.2926
FE	360	0.1140	0.0324	0.0359	0.2038
Open	360	0.3949	0.4161	0.0477	3.7303
Urban	360	0.5696	0.1265	0.2989	0.8960
lnRGDP	360	10.7019	0.5018	9.3030	12.0130
lnDis	360	6.0891	1.5661	0.6931	8.9084

**Table 5:** Correlation matrix of variables.

Variables	AGTFP	lnFin	IS	FE	Open	Urban	lnRGDP	lnDis
AGTFP	1.0000							
lnFin	0.0842 *	1.0000						
IS	−0.4498 *	−0.1537 *	1.0000					
FE	−0.5582 *	−0.0008	0.7011 *	1.0000				
Open	0.4399 *	−0.1856 *	−0.3585 *	−0.5143 *	1.0000			
Urban	0.6315 *	−0.0253	−0.6728 *	−0.6712 *	0.6810 *	1.0000		
lnRGDP	0.6180 *	0.3249 *	−0.6529 *	−0.5440 *	0.5601 *	0.8685 *	1.0000	
lnDis	−0.4119 *	0.3320 *	0.4729 *	0.4854 *	−0.6420 *	−0.6883 *	−0.5913 *	1.0000

* represents the significance at 5%.

**Table 6:** The Global Moran’s *I* of AGTFP.

Year	Moran’s *I*	Z-Value	Year	Moran’s *I*	Z-Value
2009	0.177 **	2.004	2015	0.209 ***	2.348
2010	0.207 **	2.284	2016	0.214 ***	2.375
2011	0.224 ***	2.445	2017	0.207 ***	2.347
2012	0.211 **	2.317	2018	0.174 **	2.076
2013	0.228 ***	2.485	2019	0.172 **	2.205
2014	0.172 **	1.967	2020	0.124 **	1.783

**, *** represent the significance at 5% and 1%, respectively.

**Table 7:** The results of LR and Wald tests.

	*W*_1_	*W*_2_	*W*_3_
LR SAR	57.43 (0.000)	74.95 (0.000)	38.32 (0.000)
LR SEM	56.29 (0.000)	72.67 (0.000)	33.92 (0.000)
Wald SAR	62.68 (0.000)	83.68 (0.000)	40.65 (0.000)
Wald SEM	32.74 (0.000)	13.85 (0.000)	18.17 (0.000) ^1^

^1^*p*-value in the parentheses.

**Table 8:** The results of benchmark regression.

	(1) OLS-FE	(2) OLS-RE	(3) SAR	(4) SEM	(5) SDM
			*W*_1_	*W*_1_	*W*_1_
lnFin	0.0528 **	0.0119	0.0555 ***	0.0456 **	0.0461 *
	(2.486)	(0.767)	(2.774)	(2.416)	(1.726)
IS	0.1623	0.1233	0.1482	0.0874	0.2687
	(0.888)	(0.692)	(0.862)	(0.511)	(1.638)
FE	0.0077	−0.1483	0.0102	0.0077	0.0211
	(0.029)	(−0.582)	(0.041)	(0.033)	(0.084)
Open	0.0499 ***	0.0488 ***	0.0565 ***	0.0578 ***	0.0073
	(3.498)	(3.399)	(4.097)	(4.280)	(0.489)
Urban	−0.5316 **	−0.0736	−0.5262 ***	−0.4689 **	−0.7596 ***
	(−2.491)	(−0.436)	(−2.625)	(−2.450)	(−3.418)
lnRGDP	0.1103 ***	0.1180 ***	0.1230 ***	0.1112 ***	0.0304
	(3.311)	(3.630)	(3.856)	(3.663)	(0.831)
lnDis	−0.0096 *	−0.0080	−0.0098 **	−0.0085 *	−0.0017 *
	(−1.810)	(−1.509)	(−1.970)	(−1.745)	(−1.849)
*W* × lnFin					−0.1688 ***
					(−3.958)
*W* × IS					−0.8282 **
					(−2.308)
*W* × FE					−0.4434
					(−1.170)
*W* × Open					0.0948 **
					(2.556)
*W* × Urban					0.6206
					(1.439)
*W* × lnRGDP					0.0040
					(0.063)
*W* × lnDis					0.0040
					(0.454)
Constant	−0.4153 *	−0.3952 *			
	(−1.854)	(−1.758)			
*ρ*/*λ*			0.1448 **	0.1779 **	0.4217 ***
			(2.041)	(2.309)	(5.640)
*R* ^2^	0.4031	0.3895	0.4018	0.1015	0.4780
Fixed effect	YES	YES	YES	YES	YES
Time effect	YES	YES	YES	YES	YES
Log-likelihood			540.9060	541.4749	586.7417 ^1^

^1^ z-statistics in the parentheses; *, **, *** represent the significance at 10%, 5%, and 1%, respectively.

**Table 9:** The results of direct effect, indirect effect, and total effect.

	(1) Direct Effect	(2) Indirect Effect	(3) Total Effect
lnFin	0.0853 *** (2.873)	−0.0589 * (−1.725)	0.0263 (1.239)
IS	0.3443 ** (2.120)	−0.8614 *** (−3.561)	−0.5171 ** (−2.148)
FE	0.0098 (0.037)	−0.2668 (−0.721)	−0.2569 (−0.893)
Open	0.0026 (0.170)	0.1327 *** (5.027)	0.1353 *** (6.150)
Urban	−1.1678 *** (−5.250)	1.1874 *** (3.515)	0.0196 (0.070)
lnRGDP	0.0484 (1.300)	0.0063 (0.125)	0.0547 (1.258)
lnDis	−0.0031 (−0.563)	0.0054 (0.668)	0.0023 (0.323) ^1^

^1^*p*-value in the parentheses; *, **, *** represent the significance at 10%, 5%, and 1%, respectively.

**Table 10:** The results of robustness test.

	(1) SAR	(2) SEM	(3) SDM	(4) SAR	(5) SEM	(6) SDM	(7) SDM
	*W*_2_	*W*_2_	*W*_2_	*W*_3_	*W*_3_	*W*_3_	*W*_1_
lnFin	0.0509 **	0.0406 **	0.0102	0.0535 ***	0.0440 **	0.0803 ***	0.0973 ***
	(2.494)	(2.168)	(0.485)	(2.659)	(2.300)	(2.884)	(2.703)
IS	0.1715	0.0578	0.1252	0.1530	0.1046	0.2130	0.1348
	(0.987)	(0.327)	(0.940)	(0.882)	(0.619)	(1.274)	(0.641)
FE	−0.0030	0.1117	−0.1843	0.0160	0.0505	−0.0223	0.3453
	(−0.012)	(0.459)	(−0.966)	(0.065)	(0.207)	(−0.090)	(0.588)
Open	0.0467 ***	0.0701 ***	0.0255 **	0.0523 ***	0.0608 ***	0.0303 **	0.0117
	(3.161)	(4.013)	(2.245)	(3.729)	(4.425)	(2.126)	(0.591)
Urban	−0.5422 ***	−0.4284 **	−0.4151 **	−0.5411 ***	−0.5699 ***	−0.3305	−0.7460 **
	(−2.671)	(−2.258)	(−2.394)	(−2.672)	(−3.047)	(−1.347)	(−2.501)
lnRGDP	0.1057 ***	0.1123 ***	0.0600 **	0.1161 ***	0.1236 ***	0.0527	0.0201
	(3.235)	(3.753)	(2.088)	(3.537)	(4.089)	(1.478)	(0.435)
lnDis	−0.0095 *	−0.0092 *	−0.0029	−0.0099 **	−0.0116 **	−0.0100 **	−0.0047
	(−1.886)	(−1.881)	(−0.779)	(−1.963)	(−2.288)	(−2.065)	(−0.705)
*W* × lnFin			−1.0470 ***			−0.0550	−0.0883 *
			(−8.852)			(−1.382)	(−1.926)
*W* × IS			−1.7444 *			−0.5254	1.1234 ***
			(−1.694)			(−1.596)	(3.057)
*W* × FE			−0.9072			0.3580	−1.8026 *
			(−0.803)			(0.652)	(−1.958)
*W* × Open			0.3103 ***			0.0861 ***	0.1312 ***
			(3.460)			(3.782)	(3.328)
*W* × Urban			0.7778			−1.1428 ***	1.7918 ***
			(0.742)			(−2.578)	(3.740)
*W* × lnRGDP			−0.1496			0.2077 ***	−0.0087
			(−0.695)			(3.305)	(−0.120)
*W* × lnDis			−0.0333			−0.0163 *	−0.0025
			(−1.520)			(−1.662)	(−0.210)
*ρ*/*λ*	1.3474 ***	2.1561 ***	2.2147 ***	0.0503	0.2108 **	0.1801 **	0.1631 **
	(6.038)	(8.815)	(11.037)	(0.635)	(2.225)	(2.114)	(2.185)
*R* ^2^	0.4053	0.3978	0.4777	0.4005	0.4009	0.4567	0.3725
Fixed effect	YES	YES	YES	YES	YES	YES	YES
Time effect	YES	YES	YES	YES	YES	YES	YES
Log-likelihood	583.9626	540.1004	576.4355	539.0277	541.2299	558.1885 ^1^	475.9818

^1^ z-statistics in the parentheses; *, **, *** represent the significance at 10%, 5%, and 1%, respectively.

**Table 11:** The results of regional heterogeneity test.

	(1) Eastern Region	(2) Central Region	(3) Western Region
lnFin	0.1847 ***	0.1331 ***	0.0174
	(3.825)	(7.057)	(0.370)
*W* × lnFin	−0.6269 ***	−0.0506	−0.5576 ***
	(−3.917)	(−0.453)	(−3.547)
Control variables	YES	YES	YES
*ρ*	0.9430 ***	1.2343 ***	1.5090 ***
	(4.769)	(4.954)	(10.638)
*R* ^2^	0.3835	0.0865	0.2084
Fixed effect	YES	YES	YES
Time effect	YES	YES	YES
Log-likelihood	243.3372	331.0586	212.4441 ^1^

^1^ z-statistics in the parentheses; *** represent the significance at 1%.

**Table 12:** The results of temporal heterogeneity test.

	(1) Lag 1 Period	(2) Lag 2 Period
lnFin	0.0436 **	0.0893 **
	(1.971)	(2.118)
*W* × lnFin	−1.1209 ***	−0.0399
	(−7.959)	(−1.249)
Control variables	YES	YES
*ρ*	0.4061 ***	0.2209 ***
	(−12.149)	(2.941)
*R* ^2^	0.2379	0.4293
Fixed effect	YES	YES
Time effect	YES	YES
Log-likelihood	601.8594	484.8800

**, *** represent the significance at 5% and 1%, respectively.

## Data Availability

The data used to support the findings of this study are available from the corresponding author upon request.
